# Evaluation of Distributed Acoustic Sensing (DAS) Systems with Polarization Diversity for Railway Infrastructure: Effects of Fiber Type and Installation Conditions on System Performance †

**DOI:** 10.3390/s26206463

**Published:** 2026-10-13

**Authors:** Alex Chedid, Ali Kabalan, José Grand, Tarik Hammi, Gabriel Papaiz Garbini, Ianis Gaudot, Adnand Bitri, François Bretaudeau, Elie Awwad, Renaud Gabet

**Affiliations:** 1 LTCI, Télécom Paris, Institut Polytechnique de Paris, 91120 Palaiseau, France; alexchedid1996@gmail.com (A.C.); elie.awwad@telecom-paris.fr (E.A.); 2 SNCF Réseau, 6 Avenue François Mitterrand, 93574 La Plaine Saint-Denis, France; ext.ali.kabalan@reseau.sncf.fr (A.K.); joseph.grand@sncf.fr (J.G.); ext.tarik.hammi@reseau.sncf.fr (T.H.); gabriel.papaiz@reseau.sncf.fr (G.P.G.); 3 BRGM, 3 av. Claude-Guillemin, BP 36009, 45060 Orléans Cedex 2, France; i.gaudot@brgm.fr (I.G.); a.bitri@brgm.fr (A.B.); f.bretaudeau@brgm.fr (F.B.)

**Keywords:** optical fiber, distributed acoustic sensing, coherent detection, Φ-OTDR

## Abstract

Distributed Acoustic Sensing (DAS) systems based on ϕ-OTDR are increasingly used for real-time infrastructure monitoring, but their performance is limited by coherent and polarization fading, which create signal blind spots along the fiber. This study evaluates two coherent-detection DAS architectures—with and without polarization diversity—together with the influence of optical cable type and ground-coupling method, in an operational railway environment. Field experiments were conducted along a 40 km SNCF (Société Nationale des Chemins de fer Français) railway line using train passages as excitation sources, complemented by controlled sledgehammer tap tests on a 500 m calibration platform connecting four fiber configurations (telecom vs. sensing cables, buried vs. duct-installed). Signal amplitude, differential phase, and phase Median Absolute Deviation (MAD) were used to quantify sensing performance. Polarization diversity reduced the fraction of faded signals below −40dB from approximately ≈8.4% to ≈1% of the fiber length and improved the Phase Stability Index (PSI) from ≈6dB to ≈10dB at 20 km, significantly mitigating polarization-induced signal dropouts across the 40 km span. Among the tested cables, the tight-buffered sensing fiber directly buried in the ground consistently provided the strongest and most stable response, while telecom fiber installed in a High-Density Polyethylene (HDPE) duct showed the weakest coupling. These results demonstrate that combining polarization-diversity detection with an optimized fiber-cable coupling strategy substantially improves the robustness of DAS for railway infrastructure monitoring.

## 1. Introduction

Distributed Acoustic Sensing (DAS) has revolutionized real-time monitoring by transforming standard optical fibers into dense, acoustic sensor arrays. The Rayleigh backscattered coherent light interference is analyzed to identify acoustic disturbances along the fiber optic (FO) cable. Any external event, such as ground vibrations, induces subtle phase shifts and intensity variations, enabling precise localization and continuous monitoring of these events over long distances [1].

Due to its ability to provide distributed, real-time acoustic data, DAS has become a valuable tool for various applications, such as oil and gas pipeline integrity assessment [2], security and perimeter surveillance [3], structural health monitoring [4], and traffic monitoring (e.g., road and rail vehicles) [5]. Beyond its conventional applications, DAS is also gaining traction in geophysics, particularly for seismic monitoring and subsurface imaging [6]. By leveraging existing fiber networks as seismic sensors, DAS enables the study of ground motion, fluid migration, and environmental changes with high spatial resolution and cost efficiency [7]. Its ability to operate over large distances without requiring the deployment of dedicated field equipment makes it an attractive alternative for microseismic reservoir monitoring and infrastructure health assessment.

With growing demand across multiple sectors, the DAS market is expected to see significant expansion in the coming years. Industry reports predict a compound annual growth rate (CAGR) of 10.9% between 2024 and 2030 [8], with some estimates going as high as 12.9% between 2025 and 2032 [9]. These numbers highlight the increasing recognition of DAS as a key technology for large-scale, real-time sensing systems. DAS systems primarily rely on Phase-Optical Time Domain Reflectometry (ϕ-OTDR), which detects phase variations in Rayleigh backscattering, and Optical Frequency Domain Reflectometry (OFDR), the latter often used for high-resolution measurements over shorter ranges [10]. Despite their precision, these common systems are highly susceptible to fading phenomena—specifically coherent fading and polarization fading—which can create “blind spots” along the fiber where the acoustic signal becomes temporarily undetectable or severely degraded. Coherent fading occurs when light waves backscattered from different parts of a fiber section interfere destructively. Because Rayleigh scattering centers are randomly distributed, their phases can cancel each other out. Polarization fading happens when the polarization state of the backscattered light becomes orthogonal to the receiver’s detection axis. Since the fiber randomly rotates polarization due to mechanical or thermal stress, the signal may vanish simply because the detector can no longer detect the orientation of the wave. These two phenomena lead to a drop in signal intensity below the detection threshold, resulting in the aforementioned blind spots. To address these issues, various solutions have been explored, including frequency modulation techniques [11] and more advanced receiver designs, such as dual-polarization coherent detection [12]. These works aim to enhance signal stability and improve detection sensitivity, making DAS more reliable for a wide range of applications.

While the potential of DAS for monitoring urban traffic, human activity, and seismic events across deployed optical infrastructures has been successfully demonstrated [13,14,15,16,17], these approaches typically face operational constraints in terms of acoustic bandwidth, measurement sensitivity, maximum reach, and deployment overhead. Polarization diversity techniques [12] and specialized cable designs have been separately explored in the literature, yet their joint impact and practical trade-offs under real operational environments remain insufficiently documented [18,19]. From an industrial perspective, deploying dedicated sensing cables or upgrading interrogators with polarization-diversity modules introduces significant financial and hardware complexity. It is therefore crucial to determine whether existing telecom fiber networks are sufficient for operational monitoring, and to what extent polarization diversity compensates for sub-optimal cable types or installation conditions (such as direct burial versus conduit housing). This study addresses this gap by presenting a comprehensive field evaluation along an active railway track in France. We systematically assess the interplay between interrogator architecture (ϕ-OTDR with vs. without polarization diversity) and four distinct fiber configurations subjected to complex train-induced acoustic signals. The primary objective is to quantify the real-world performance gain of polarization diversity across these deployment scenarios, providing clear insights into the techno-economic trade-offs between hardware complexity and cable selection for industrial DAS monitoring.

The article is organized as follows: Section 2 presents a comparative analysis of two coherent detection DAS architectures with and without polarization diversity and characterizes the experimental field site and calibration site configuration, specifically detailing the different types of optical fibers used and their locations relative to the railway line. Section 3 compares the capabilities of both detection configurations, providing insights into their technical limitations and evaluating sensing performance based on field experiments (using train passages as a reference). Section 4 presents a more detailed analysis, examining different types of optical cables and their impact on DAS sensitivity, with the aim of identifying which cable type offers the best signal quality and sensitivity for detecting events.

Finally, the article concludes with a summary of key findings, discussing their broader implications and potential avenues for future research. This manuscript is an extended version of our conference paper published in the SPIE Proceedings [20] (DOI: https://doi.org/10.1117/12.3062236). It provides substantial new analyses, updated datasets, and deeper physical insights, with newly added content constituting significantly more than 50% of the original work. While the initial conference paper focused solely on a single cable type and evaluated the preliminary benefits of polarization diversity, this work vastly expands upon those findings. Specifically, we present entirely new experimental datasets, introduce new statistical metrics—including the Median Absolute Deviation (MAD)—and deliver a novel comparative analysis evaluating the impact of different fiber-optic cable types and burial/installation configurations.

## 2. Distributed Acoustic Sensing Systems: Experimental Setups and Evaluation Approach

### 2.1. Experimental Setups

**Figure 1:**
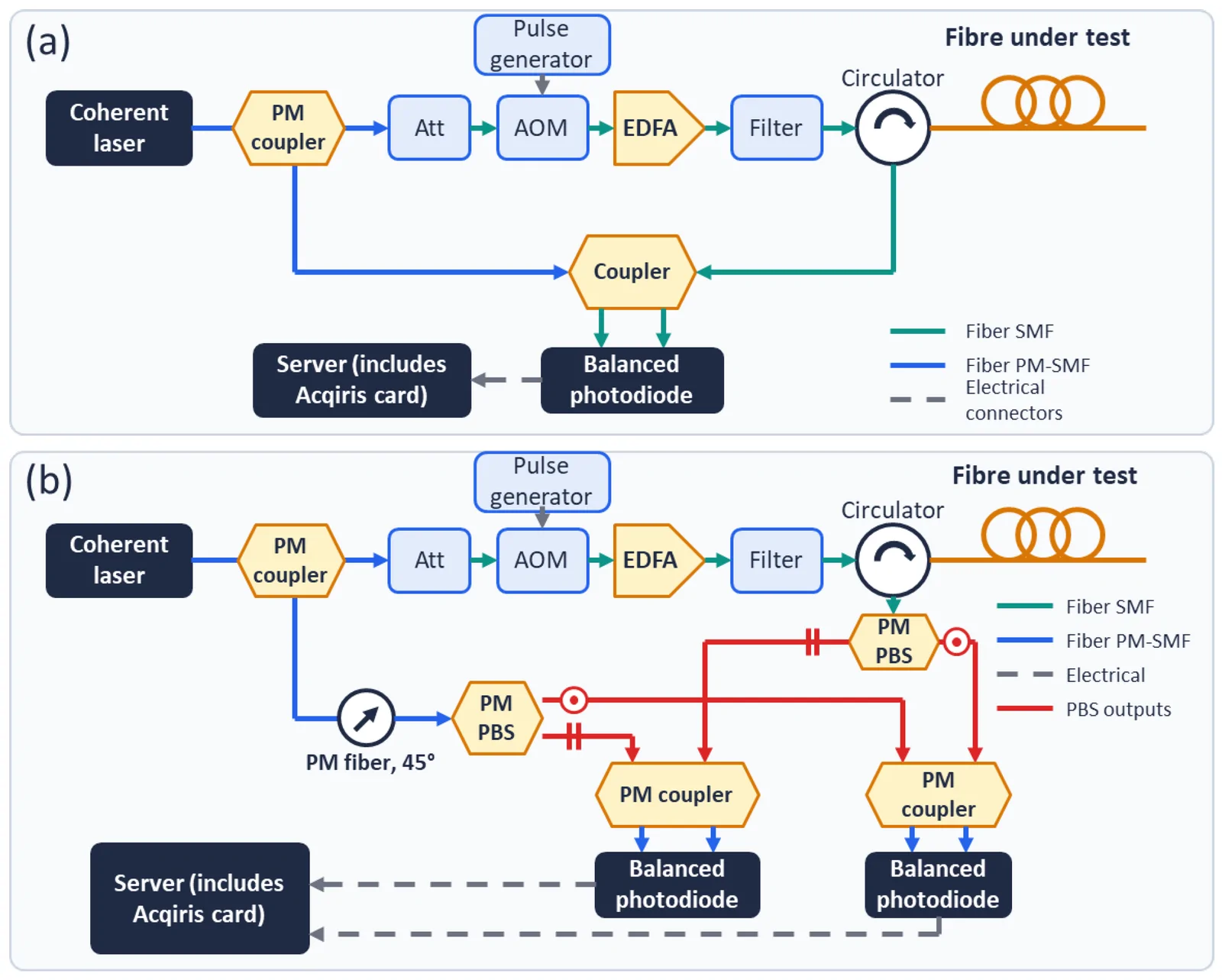
Distributed acoustic sensing DAS systems: (**a**) Without polarization diversity. (**b**) With polarization diversity (reproduced from [20] under SPIE author self-reuse guidelines).

As outlined in the introduction, we compare two DAS configurations: one implementing polarization diversity (see Figure 1b) and another without it (see Figure 1a). Both systems employ a narrow-linewidth laser operating at a central wavelength of 1550 nm to ensure a sufficiently long coherence length (Δν<∼100Hz). Except for the fibers under test—which are detailed in Section 2.2—all internal fiberized components are standard commercial off-the-shelf devices using standard single-mode fibers (SMF-28), either isotropic or polarization-maintaining depending on the optical path constraints. An acousto-optic modulator (AOM) shapes the optical signal into pulses while applying a frequency shift Δf. The Pulse Repetition Rate (PRF) is set to 1.6kHz, ensuring a maximum unambiguous sensing range of over 60km and preventing any overlap of the backscattered signal along the 40km sensing link. The optical pulses are amplified at the fiber input using an Erbium-Doped Fiber Amplifier (EDFA), operated in the saturation regime to ensure gain stability and tuned to reach the threshold power of non-linear optical effects at the fiber input, with a bandpass filter inserted to suppress amplified spontaneous emission noise. The amplified signal is injected into the sensing fiber via a circulator, and the distributed Rayleigh backscatter signal is collected. To enable a direct and unbiased comparison, both interrogator setups share the same master laser source via an optical coupler. This configuration eliminates uncorrelated laser phase fluctuations between the two systems, ensuring that performance differences stem solely from the polarization-diversity detection scheme. The experimental setup and acquisition campaign were previously described in [20]. In this study, a different dataset recorded under the same experimental conditions is analyzed.

In both setups, the backscattered light is mixed with a local oscillator (LO) and converted to an electrical signal through a balanced photodiode. The resulting DAS signal is centered at an intermediate frequency of approximately Δf=55MHz in the non-polarized system and Δf=69MHz in the polarization diversity system; this difference arises from variations in the AOMs used. With a 250 MS/s sampling rate ($f_{\mathrm{Nyquist}}=125\;\mathrm{MHz}$), the ≈40MHz spectrum of the 30 ns pulse remains fully within the Nyquist band for both 55 MHz and 69 MHz operations, thereby enabling alias-free demodulation. These intermediate frequencies correspond to off-the-shelf commercial acousto-optic modulators with equivalent specifications (e.g., rise time and insertion loss) available in our laboratory, with no observed difference in signal quality between the two configurations. The primary distinction between the two configurations is that the polarization diversity architecture splits both the backscattered light and the LO into horizontal and vertical polarization components, requiring two balanced photodetectors instead of one. Whereas the polarization state evolves unpredictably along standard sensing fibers, traditional architectures suffer from random polarization fading whenever the backscattered signal becomes orthogonal to the local oscillator. Conversely, the polarization-diversity configuration utilizes polarization-maintaining components in the optical receiver to process two orthogonal polarization states simultaneously, ensuring continuous signal recovery along the entire sensing link.

### 2.2. Field Access and Calibration Site Configuration

**Figure 2:**
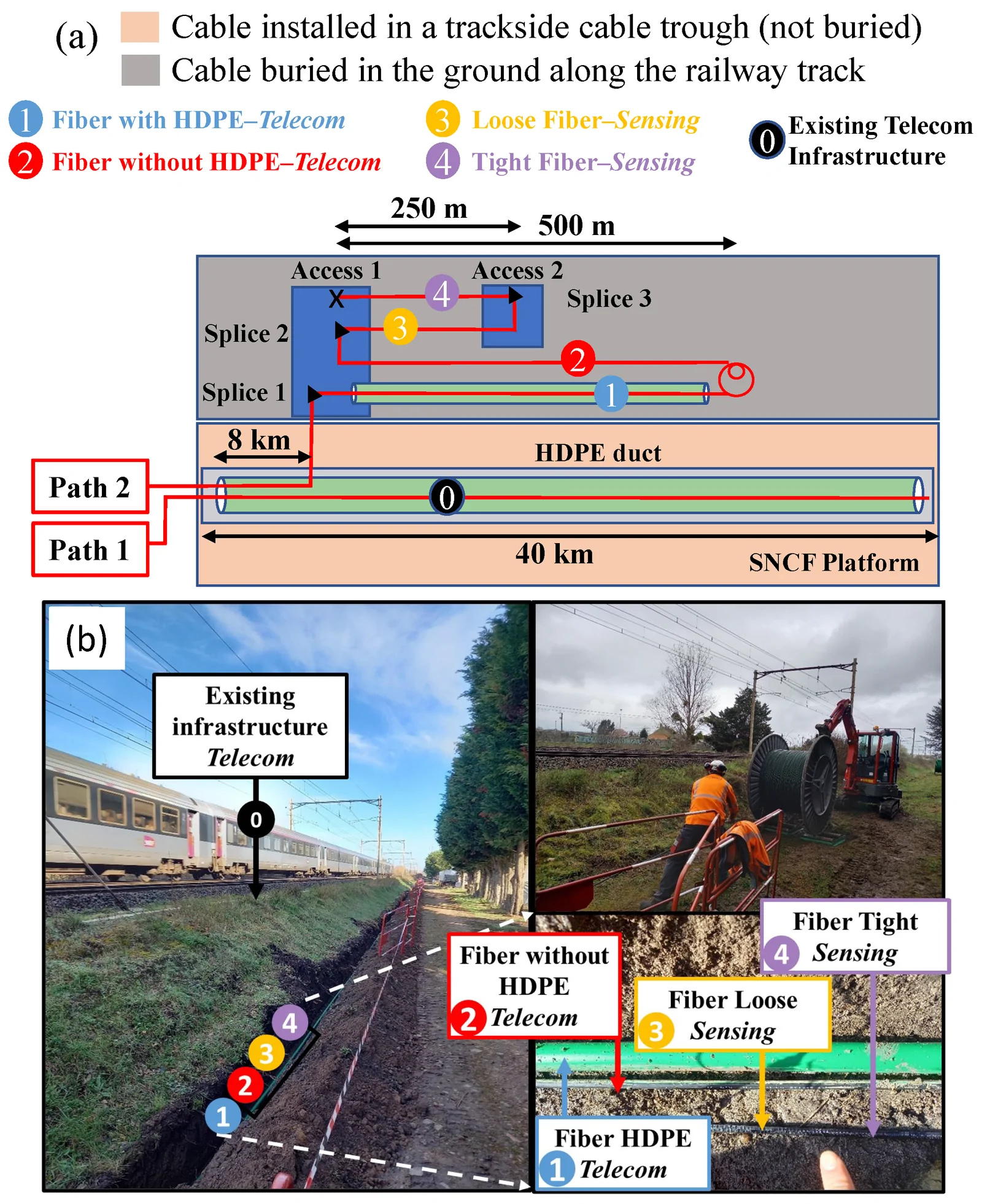
(**a**) Diagram of the SNCF experimental platform illustrating two accessible optical paths: a 40 km linear fiber path, and a non-linear path incorporating a calibration platform that provides access to four connected fibers, each featuring a different cable-to-ground coupling configuration. (**b**) Illustration of the setup installed on the calibration platform, showing the various cables and coupling conditions.

Within the framework of the ANR SITcomOptics project, a 40 km pilot railway section has been made available through a partnership with Société Nationale des Chemins de fer Français (SNCF). Additionally, a dedicated 500 m calibration platform was established to interconnect optical fibers from various cable coupling types with an existing telecom cable (Figure 2).

Figure 2a presents a schematic of the field experimental setup, highlighting the two most representative optical paths. Path 1 consists of a 40 km linear fiber section, predominantly installed in a trackside cable trough (marker (0)). Path 2 follows a non-linear route incorporating the 500 m calibration platform, which provides access to four fibers, each representing a distinct combination of cable type and ground coupling method ((1) to (4) markers). In the diagram, grey-shaded areas indicate cable segments directly buried along the track, while orange-shaded areas correspond to fibers installed in a trackside cable trough. Access points (Splices 1–3) enable reconfiguration and selective interrogation of the fibers. The two remaining optical paths, although deployed in a similar manner (along nearly the same route using different fibers for simultaneous interrogation by different DAS systems), are omitted from the figure to maintain visual clarity.

In the calibration platform, two main cable types were deployed: a 72-fiber loose-tube telecom cable used by SNCF Réseau, and a 2-fiber cable available in both loose-tube and tight-buffered configurations used by BRGM for sensing. Phase-sensitive DAS measures Rayleigh backscatter phase shifts induced by strain (Δϵ) and temperature (ΔT). While slow thermal fluctuations cause low-frequency phase drift, dynamic railway monitoring strictly relies on fast strain variations. Tight-buffered fibers feature a protective outer jacket directly bonded to the cladding, ensuring high mechanical coupling and direct strain transfer. Conversely, loose-tube fibers house the glass strain-free inside a gel-filled buffer tube; this structural design isolates the fiber from ambient thermal expansion, but creates mechanical decoupling that dampens dynamic strain transfer and reduces signal fidelity under train passages. Two distinct soil coupling methods were implemented: direct burial in a micro-trench at a depth of 40 cm, and installation inside a High-Density Polyethylene (HDPE) duct (Figure 2b).

The four connected fibers (all buried along the railway track) therefore correspond to the following combinations of cable type and coupling method:

- Fiber With HDPE–*Telecom*: Fiber from the 72-fiber loose-tube telecom cable, installed within an HDPE-sheathed cable configuration.
- Fiber Without HDPE–*Telecom*: Fiber from the same 72-fiber cable, directly installed in a micro-trench.
- Fiber Loose–*Sensing*: Fiber from a 2-fiber loose-tube cable, directly installed in a micro-trench.
- Fiber Tight–*Sensing*: Fiber from a 2-fiber tight-tube cable, directly installed in a micro-trench.

## 3. Evaluating DAS Performances: With vs. Without Polarization Diversity

The complex optical field is reconstructed from the real-valued recorded signals using the standard Hilbert transform via Fast Fourier Transform (FFT) and negative frequency filtering [10].

### 3.1. Amplitude Evaluation

**Figure 3:**
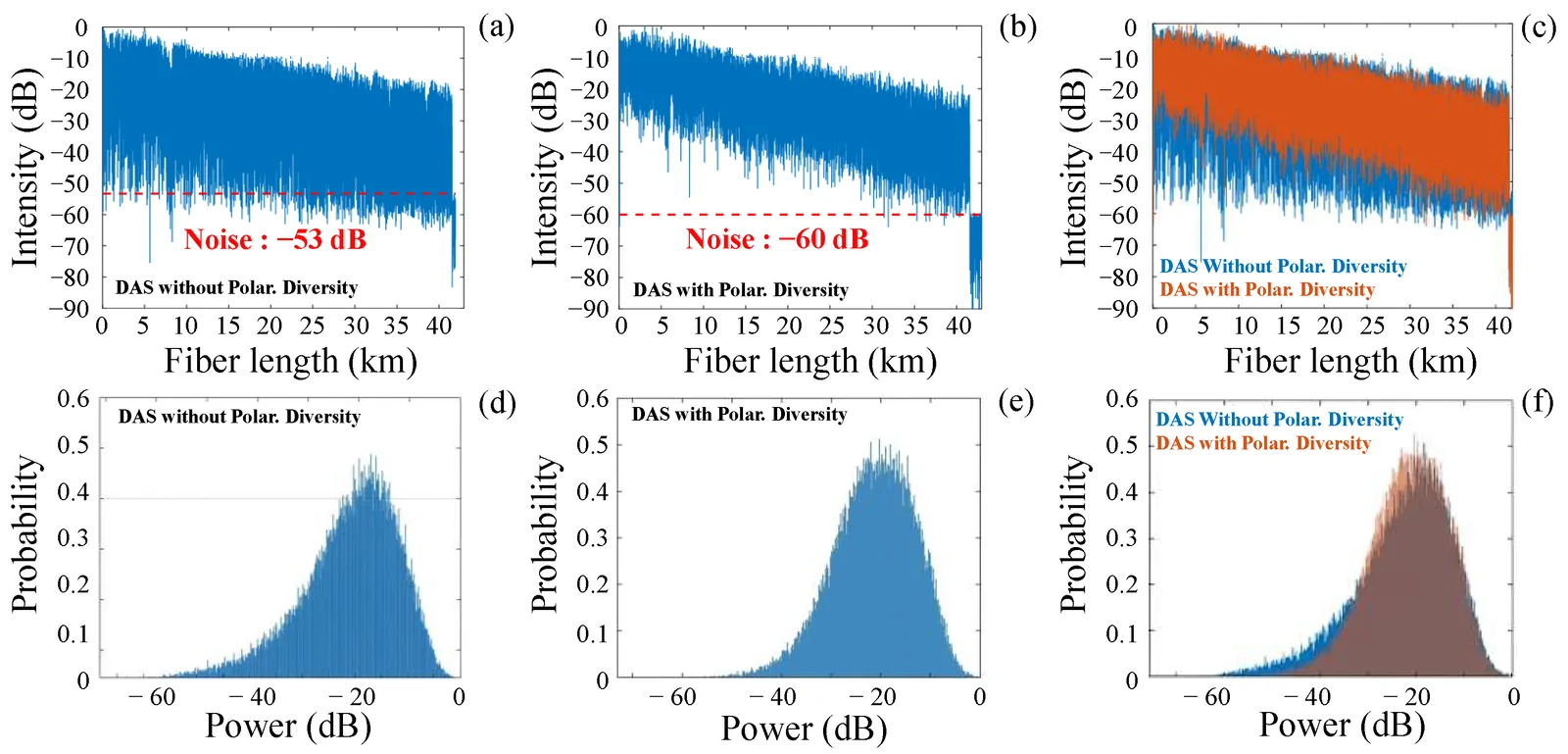
Impact of polarization diversity on Distributed Acoustic Sensing (DAS) signal characteristics over a 40 km fiber span. (**a**) Normalized beat signal intensity for DAS without polarization diversity. (**b**) Normalized beat signal intensity for DAS with polarization diversity. (**c**) Comparative overlay of beat signal intensities from (**a**,**b**). (**d**) Histogram of distributed signal power for DAS without polarization diversity. (**e**) Histogram of distributed signal power for DAS with polarization diversity. (**f**) Comparative histogram of distributed signal power from (**d**,**e**).

To evaluate the performance of the two DAS systems, two fibers from a 72-fiber loose-tube telecom cable (Fiber_path_1), each spanning 40 km, were used. The backscattered single-shot signals detected by each system under quiet, train-free conditions were examined using a 30 ns pulse width to highlight differences in signal quality and are presented in Figure 3. Although the two DAS systems interrogated adjacent fibers within the same cable, both channels exhibited identical attenuation profiles and equivalent baseline signal contrasts, ensuring that polarization diversity remained the governing variable while spatial fading locations naturally differed randomly.

We can see a clear difference between the backscattered light signals recorded by the two DAS configurations. The system operating without polarization diversity exhibits large-intensity variations along the fiber, with signal levels often dropping close to the noise floor, at around −53dB (Figure 3a). The noise floor was established from several evening measurements without train traffic (1 min at  PRF = 1.6 kHz). The majority of these signal drops originate from polarization fading. In contrast, the DAS with polarization diversity provides a more stable measurement, with signal levels remaining well above the noise floor, which is around −60dB in this case (Figure 3b). Polarization fading is significantly mitigated. Coherent fading persists, as its mitigation is beyond the scope of this study.

To further quantify this observation, the spatial power distribution along the 40km fiber was analyzed via the histograms in Figure 3d–f, corresponding to an instantaneous single-shot acquisition in undisturbed conditions. To evaluate fading occurrence on an absolute physical basis, a common signal exploitability threshold was set at −40dB, representing the minimum backscattered power required by the demodulation chain to reliably recover phase information regardless of the baseline noise floor.

For the standard setup, approximately 8.4% of the spatial points fall below this −40dB threshold (spanning down to the −53dB noise floor). In contrast, with polarization diversity, only ∼1% of the backscattered signal drops below −40dB (spanning down to −60dB). While these percentages illustrate an instantaneous order of magnitude for spatial fading mitigation from a single-shot acquisition, they should not be interpreted as temporal or statistical averages. Instead, a comprehensive statistical evaluation over extended temporal datasets is conducted in Section 3.2 in the phase domain.

### 3.2. Phase Evaluation

**Figure 4:**
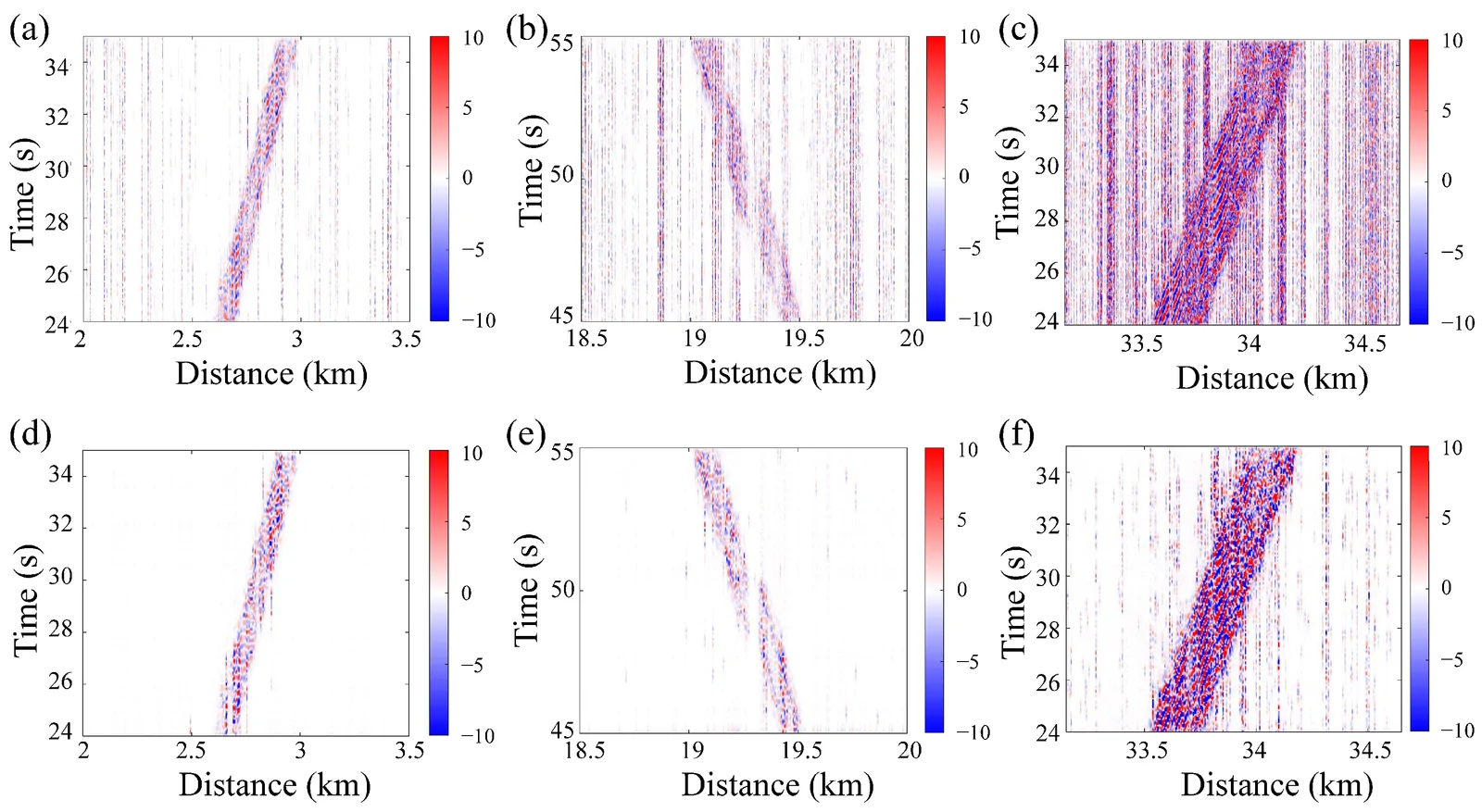
Differential phase maps (in rad) from DAS for a passing train. The top row (**a**–**c**) shows results without polarization diversity; the bottom row (**d**–**f**) shows results with polarization diversity, at three positions relative to the acquisition center (∼3 km, ∼20 km, ∼34 km). Two fibers from the same 72-fiber loose-tube telecom cable (Fiber_path_1), each spanning 40 km, were used for this analysis.

In ϕ-OTDR sensing, the differential phase is the primary metric used to quantify acoustic disturbances. It represents the phase shift of backscattered light induced by fiber strain over a specific distance, known as the gauge length (GL). While the GL determines the system’s spatial resolution and sensitivity, the differential phase provides the linear relationship needed to accurately reconstruct the frequency and amplitude of the environmental vibrations. For the phase stability analysis, a total dataset of nearly 100 train passage events recorded across the testing campaign was examined. Representative operational conditions—including short regional trains (Figure 4a,b,d,e) and long multi-unit trains (Figure 4c,f)—were selected to cover operational conditions along the 40km link. To ensure a rigorous and reproducible comparison, the evaluation was performed on identical train passage events recorded simultaneously by both systems (with and without polarization diversity), using a GL of 6 m. Individual records of trains passing at different positions were selected to illustrate local phase behavior, while the quantitative metrics were statistically evaluated across the full dataset.

As illustrated in Figure 4a–c for the system without polarization diversity, signal quality degrades progressively with fiber length. At short distances (Figure 4a, ∼3 km from the acquisition unit), the train passage is clearly visible as continuous phase fringes with minimal background noise. At intermediate distances (Figure 4b, ∼20 km), increased background fluctuations and reduced fringe contrast indicate a lower signal-to-noise ratio (SNR). At the far end of the fiber (Figure 4c, ∼34 km), the phase map shows significant noise, with fragmented or fading fringes, making the train signal less distinguishable. This degradation is attributed to optical attenuation of the Rayleigh backscatter over long distances, lowering the SNR. Consequently, as the optical power drops, a significantly higher number of locations fall below the detection threshold, drastically increasing the number of blind spots. This effect is further compounded by phase noise accumulation, dispersion, and environmental factors (e.g., temperature variations).

By contrast, Figure 4d–f show the results obtained with polarization diversity. The phase fringes remain sharper and more continuous at all distances, particularly at 20 km and 34 km, where the non-diversity system suffers from severe fading. The improvement is especially evident in Figure 4f, where polarization diversity mitigates the polarization fading effect, preserving the visibility of the train signature even in the high-loss regime.

To quantify the variability of the differential phase response, a robust statistical measure was used. The Median Absolute Deviation (MAD) provides a reliable assessment of phase stability and uniformity along the fiber. This approach effectively differentiates regions of consistent phase behavior from those exhibiting significant fluctuations, which may result from environmental factors, external disturbances, or limitations inherent to the DAS technique. Being inherently resilient to outliers, the MAD offers a precise evaluation of phase coherence, making it particularly suited for analyzing signal integrity in noisy or dynamically changing conditions [21,22]. In the context of ϕ-OTDR, a low and constant MAD value along the fiber indicates high phase stability and a uniform noise floor. Consequently, comparing the MAD profiles of the two systems allows us to quantify how effectively polarization diversity mitigates signal fading and homogenizes phase stability across the 40 km range prior to entering the attenuation-limited regime.

MAD is computed as follows:

(1) $$\mathrm{MAD}\left(\sigma_{\mathrm{phase}\text{\_}\mathrm{MAD}}\right)=\mathrm{median}\left(\left|\varphi_{i}-\tilde{\varphi }\right|\right)$$

where $\varphi_{i}$ represents the phase value at a given position along the fiber, and $\tilde{\varphi }$ is the median phase over the GL. This metric highlights deviations from the median phase, allowing for a clear distinction between stable and fluctuating regions.

**Figure 5:**
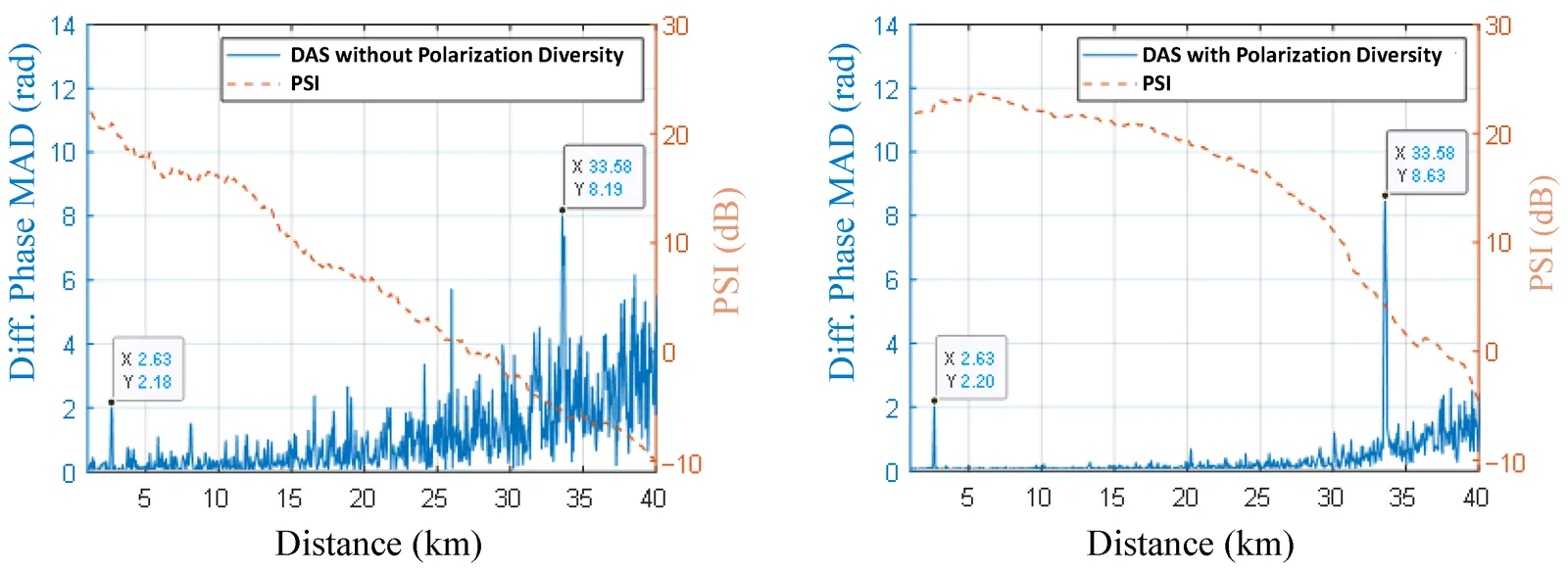
In blue: the Median Absolute Deviation (MAD) of the phase map over the entire 40 km fiber length for (**Left**) the DAS system without polarization diversity and (**Right**) the DAS system with polarization diversity. The orange curve represents the Phase Stability Index (PSI), a normalized metric indicating the baseline phase stability along the fiber. Peaks around 2 km and 33 km represent phase variations caused by the passage of two trains. Two fibers from a 72-fiber loose-tube telecom cable (Fiber_path_1), each spanning 40 km, were used for this analysis.

Figure 5 presents the differential phase MAD ($\sigma_{\mathrm{phase}\text{\_}\mathrm{MAD}}$) results for the train passages shown in Figure 4, focusing on a scenario where two trains are detected simultaneously at different positions along the fiber: one between 2 km and 3.5 km (Figure 4a) and the other between 33 km and 35 km (Figure 4c) from the acquisition unit. The MAD curves were obtained by averaging 0.5 s of recording at a pulse repetition frequency of 1.6 kHz, corresponding to 800 single-shot traces.

It should be emphasized that while the phase MAD accounts for overall statistical variability—inherently combining optoelectronic measurement noise with physical strain changes—the spatial profiles in Figure 5 clearly distinguish these two contributions. Without polarization diversity (Figure 5, left), the baseline is dominated by high-frequency fluctuations caused by random optical fading along the fiber length. When polarization diversity is enabled (Figure 5, right), this optoelectronic noise floor is suppressed down to near zero. Consequently, only the genuine physical strain events—specifically the two localized train passages at x=2.63km and x=33.58km—emerge above the quiet background with high contrast, demonstrating that the MAD reduction achieved via polarization diversity reflects a true mitigation of fading noise.

As observed in the MAD spatial profiles, polarization diversity primarily acts by lowering the baseline noise floor rather than altering peak strain amplitudes. To quantitatively assess this baseline phase stability along the fiber in the absence of local perturbations, a normalized metric referred to as the Phase Stability Index (PSI) is derived from the phase MAD background level. It is defined as:

(2) $$\mathrm{PSI}=10\log_{10}\left(\frac{\sigma_{0}^{2}}{\sigma_{\mathrm{baseline}\text{\_}\mathrm{phase}\text{\_}\mathrm{MAD}}^{2}}\right)$$

where $\sigma_{\mathrm{baseline}\text{\_}\mathrm{phase}\text{\_}\mathrm{MAD}}^{2}$ is the normalized variance of the phase MAD in undisturbed conditions along the 40 km fiber span (expressed in ${\mathrm{rad}}^{2}$), and $\sigma_{0}^{2}=1\;{\mathrm{rad}}^{2}$ is an implicit reference unitary level ensuring a dimensionless ratio inside the logarithm. A lower baseline variance directly translates into a higher PSI, reflecting a higher signal phase stability.

The MAD results are corroborated by the statistical PSI analysis performed over 1min (N≈96,000 single-shot train-free baseline traces) collected across the testing days: at a distance of 20km, polarization diversity consistently improves the average phase stability index from 6±0.9dB to 10±0.6dB. This statistically significant enhancement demonstrates a robust reduction in baseline phase noise, ensuring reliable detection performance across the 40km sensing range. From an operational perspective, suppressing these baseline fluctuations facilitates the reliable discrimination of genuine acoustic events by automated detection algorithms.

The joint analysis of the PSI and MAD profiles provides a clear assessment of the system’s measurement reliability over the 40km range. A notable observation is the peak at 33km, which appears more intense in the MAD domain than the peak at 2km, despite a significantly lower PSI. This behavior highlights the statistical nature of the MAD: at 33km, the proximity to the noise floor (PSI∼5dB) induces heightened phase uncertainty, making the measurement more susceptible to fluctuations. In contrast, the low and stable MAD baseline maintained over the first 30km demonstrates that polarization diversity effectively ensures phase stability as long as the PSI remains sufficient. Ultimately, the rise in MAD at longer distances serves as a diagnostic tool, quantifying the transition from reliable sensing to the system’s noise-limited regime.

The MAD can exceed π radians because the differential phase is an unwrapped signal, representing cumulative physical strain that often surpasses the 2π phase cycle. Furthermore, in low-PSI regions (as seen beyond 33 km), the proliferation of cycle slips and phase noise leads to high statistical dispersion, causing the MAD to rise significantly above the π threshold.

## 4. Impact of Optical Fiber Cable Design and Ground Coupling on DAS Measurement

To study the influence of different fiber optic installation on DAS system performance, sledgehammer shots were carried out near the calibration platform. For this, fiber path 2, which follows a non-linear route incorporating the 500 m calibration platform and provides access to four fibers with varying cable types and ground coupling methods, was used (as described in Section 2 and shown in Figure 2).

### 4.1. Geophysical Data Acquisition

The main purpose of the installation is to compare how different cable–soil coupling conditions as well as cable design (telecom versus sensing) affect the ability of the DAS system to retrieve meaningful geophysical information. A commonly used approach is to generate a vertical seismic impulsive source at the surface and record the resulting wavefield, which can then be analyzed to investigate the subsurface structure.

In our case, the vertical impulsive seismic source was produced using an accelerated sledgehammer (8 kg) hitting the ground. Shots were performed every 6 m along the first 380 m of the cable. The DAS acquisition parameters were identical to those used for the train measurements to ensure consistency between the two datasets.

### 4.2. Quick Overview of the Results from the Geophysical Campaign

**Figure 6:**
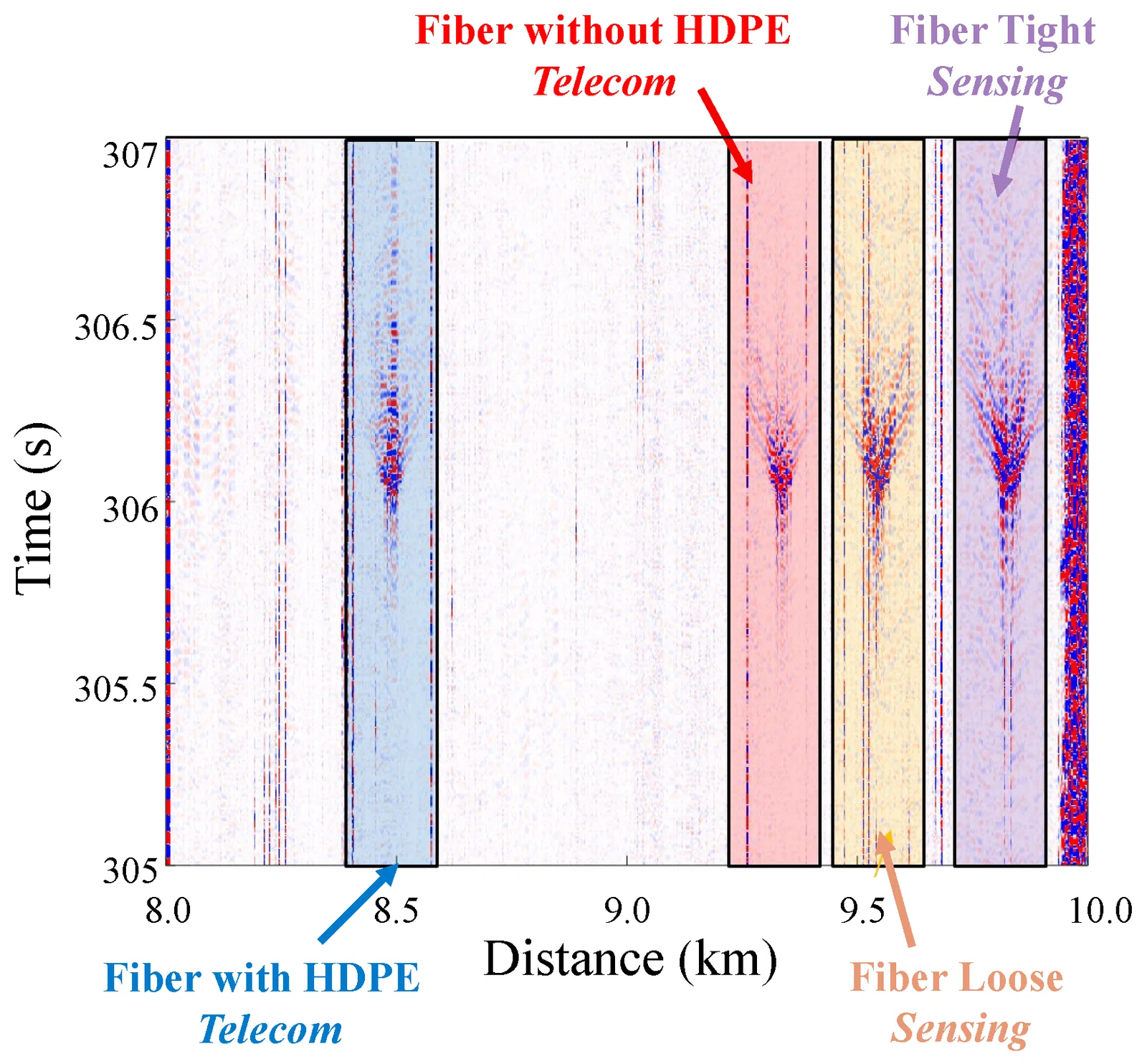
Phase map analysis in a polarization diversity DAS system: effect of a tap test on the system response using different types of optical cables (72-fiber *Telecom* and 2-fiber *Sensing*) and installation conditions (direct buried or in conduit).

**Figure 7:**
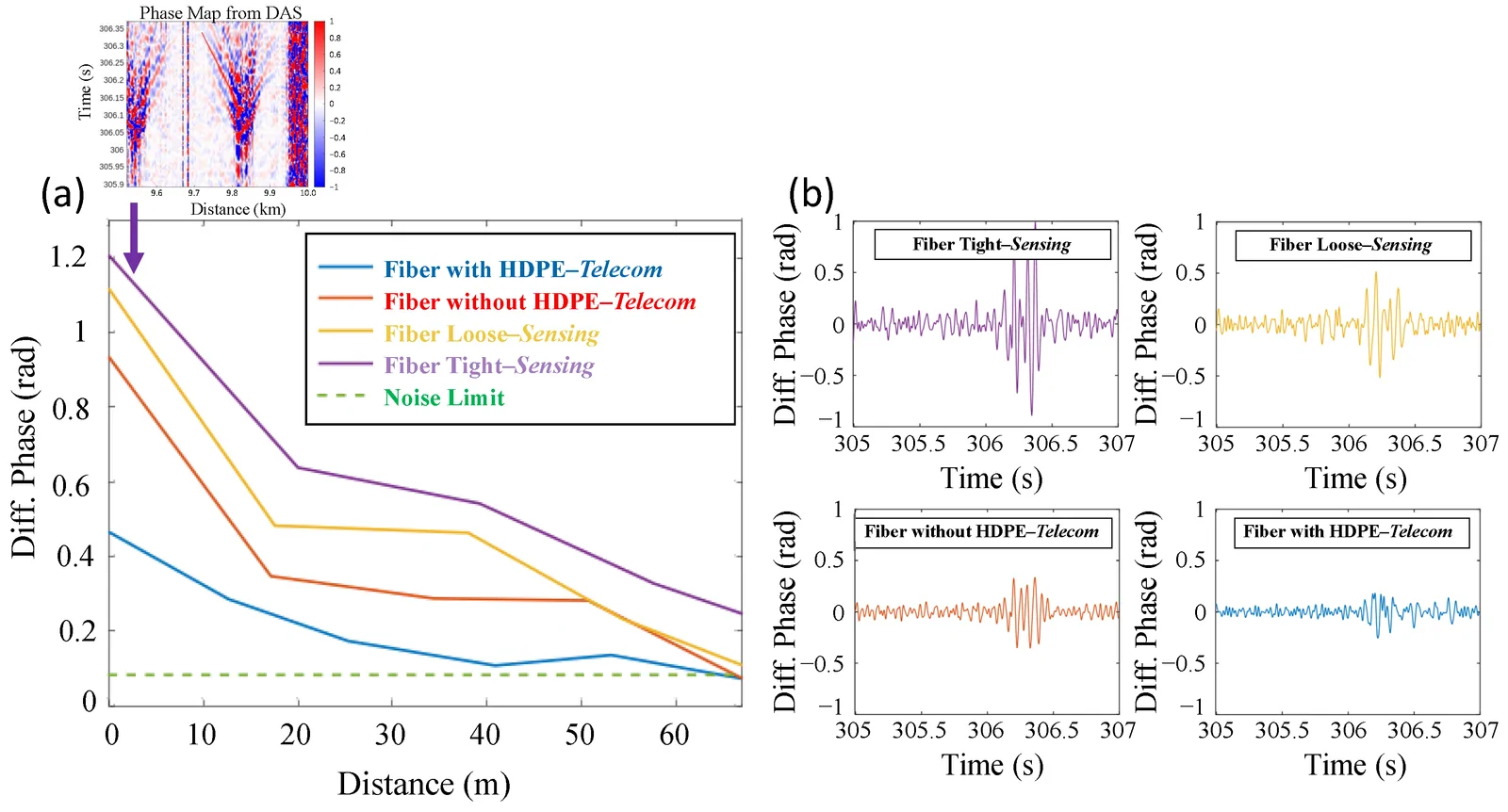
(**a**) Absolute variation of the detected signal as a function of distance, based on the slopes of the tap test signals measured by the DAS system. The zero-meter point corresponds to the center of the disturbance. (**b**) Time-domain signal (differential phase signal) measured at 30 m from the disturbance center.

**Figure 8:**
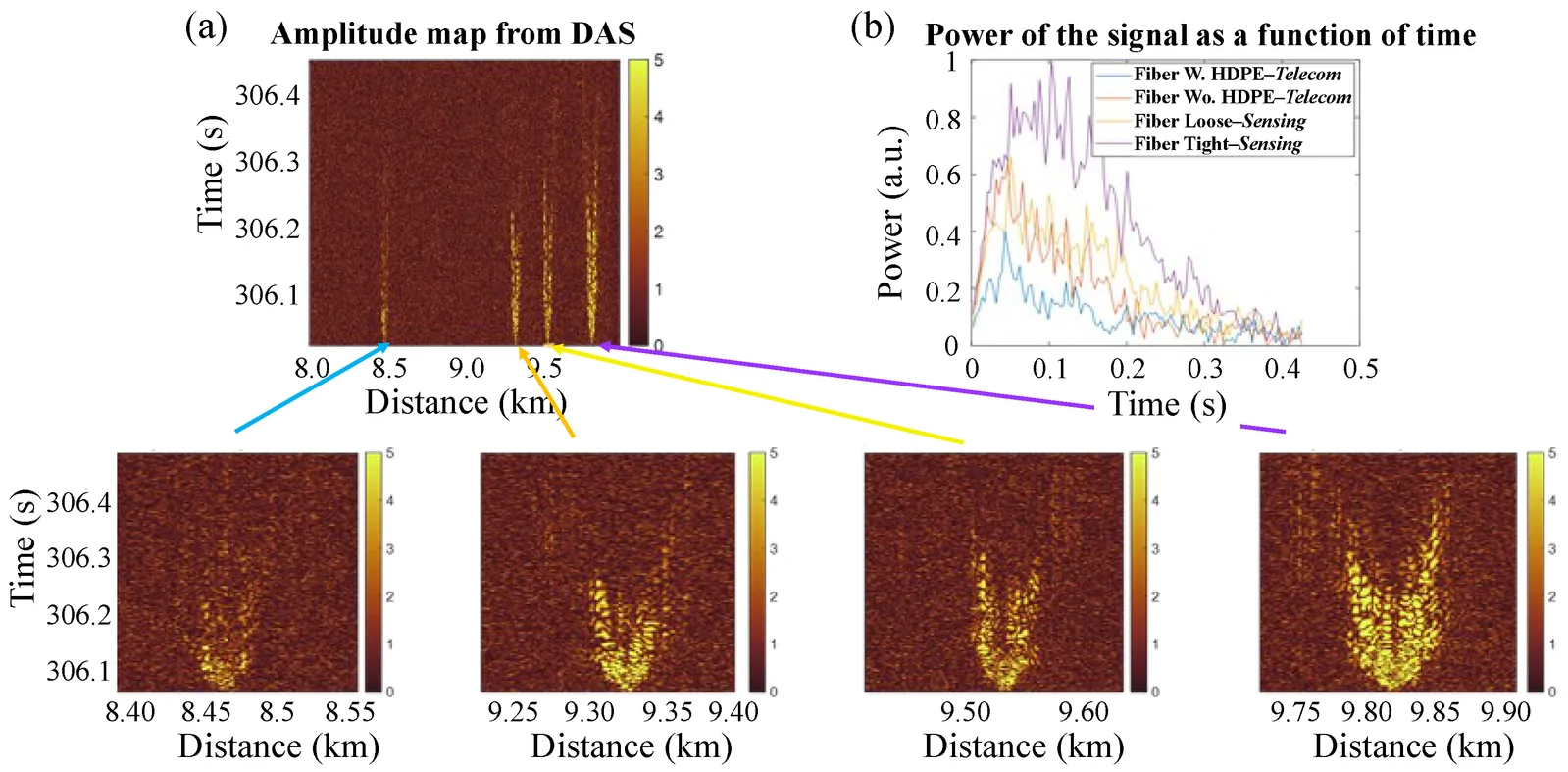
(**a**) Spatio-temporal amplitude map derived from the magnitude of the reconstructed analytic signal during a tap test; (**b**) signal power (squared magnitude) as a function of time at the impact location for the four fiber types, normalized by the maximum peak power among all configurations. Bottom: time windows used for the amplitude-based power calculation.

Figure 6 shows the phase response to a single tap test impact for the four optical fiber types: Fiber HDPE–*Telecom*, Fiber Without HDPE–*Telecom*, Fiber Loose–*Sensing*, and Fiber Tight–*Sensing*. At first glance, a clear difference in response can be observed among these fibers. Firstly, it is worth noting that all fibers are capable of detecting impacts, including those in loose tubes, despite the fact that their design is intended to decouple the fiber from environmental vibrations. However, not every fiber detects environmental variations with the same sensitivity. In particular, the Fiber Tight–*Sensing*, located between 9.75 km and 10 km, exhibits a stronger and more easily detectable signal compared to the other fiber types. A more detailed analysis will be presented next to confirm and further explore this observation (Figure 7 and Figure 8).

It is important to note that the phase traces observed on the four fibers originate from the same signal source. As illustrated in Figure 2, the fiber installation allows the same disturbance to be simultaneously detected by all four fibers, each featuring a different coupling configuration. Since differential phase is measured here, the same impact signal represented in Figure 6 at different positions is not affected by the small amplitude attenuation observed between 8.5 and 10 km. Therefore, the traces can be directly compared without requiring further normalization.

The slope of the detected signals, corresponding to the V-shaped pattern observed in Figure 6, provides key information on wave propagation in the medium, including propagation velocity, distance-dependent attenuation, and the characteristics of fiber–environment coupling. A steep slope indicates a slower propagation velocity, as the signal arrival time changes rapidly with distance, whereas a shallow slope corresponds to a faster velocity.

Figure 7 shows the absolute variation of the detected differential phase as a function of distance from the impact point, based on the slopes of the tap test signals measured by the DAS system. These results demonstrate that the signal response from the Fiber Tight–*Sensing* configuration is noticeably stronger than those from the other fiber types. Beyond ≈60 m from the disturbance center, signals from all fibers except Fiber Tight–*Sensing* decay to the baseline noise floor. This floor corresponds to the spatial asymptotic limit in Figure 7a far from the perturbation, which aligns with baseline measurements recorded without train traffic.

Additionally, the time-domain signals at 30 m from the disturbance (Figure 7b) confirm that Fiber Tight–*Sensing* directly installed in a micro-trench produces a signal with a peak phase-difference strain amplitude at least twice as large as the other fibers. Conversely, Fiber HDPE–*Telecom* exhibits the weakest signal, reaching noise level by 30 m. The noise baseline was established from several evening measurements without train traffic or tap tests.

By analyzing the amplitude of the backscattered signal (Figure 8) rather than the phase (Figure 6), a similar conclusion can be drawn. Specifically, the spatio-temporal amplitude map in Figure 8a is obtained from the magnitude of the analytic signal, reconstructed via the Fourier/Hilbert processing scheme described in the introduction of Section 3. To evaluate the transient response at the impact location for each cable configuration, the time-dependent power profiles in Figure 8b correspond to the squared magnitude extracted at the tap position, normalized by the peak maximum power of the highest-responding configuration. This relative power normalization allows for a direct comparison of both relative sensitivity and energy decay dynamics across the four cable structures on a unified scale.

This amplitude-based evaluation confirms the performance ranking observed in the phase domain: the Fiber Tight–*Sensing* directly installed in a micro-trench exhibits the highest sensitivity to the tap test event, the Fiber HDPE–*Telecom* shows the lowest, while Fiber Loose–*Sensing* and Fiber Without HDPE–*Telecom* (also directly installed in a micro-trench) display intermediate sensitivity levels.

It should be noted, however, that these experiments jointly involve both cable design and ground-coupling conditions; consequently, their individual contributions to the observed response cannot be completely decoupled.

## 5. Conclusions

This study provides a comprehensive evaluation of Distributed Acoustic Sensing (DAS) performance, focusing on two key aspects: the impact of polarization diversity in coherent detection systems, and the influence of fiber type and installation conditions on signal quality. The comparative analysis of the two DAS configurations reveals a clear performance advantage for the system integrating polarization diversity, which demonstrates improved phase stability, higher signal-to-noise ratio, and significantly reduced susceptibility to polarization fading over long distances.

Furthermore, experimental results from the geophysical seismic tests confirm the critical role of fiber coupling and the installation method in determining DAS sensitivity. Among the tested configurations, the Fiber Tight–*Sensing* directly installed in a micro-trench consistently delivered the strongest and most reproducible signals in the time-domain response. In contrast, standard telecom fibers, particularly those installed inside an HDPE duct, exhibited significantly lower sensitivity and poorer coupling to the surrounding medium. It is important to recall that cable design and ground-coupling effects were examined simultaneously, making it challenging to isolate their respective contributions to the observed response. While general DAS response capabilities and dark-fiber sensing have been documented [18,19] our empirical results highlight the real-world operational trade-offs—demonstrating that severe coupling losses in HDPE conduits can be effectively compensated for by interrogator-level polarization diversity.

Overall, this work highlights the importance of optimizing both the optical system architecture and the physical deployment of sensing fibers. Such considerations are essential not only for enhancing detection sensitivity and phase stability but also for enabling advanced seismic or structural analysis based on DAS data. Future work will explore the integration of additional diversity schemes, further characterization of coupling mechanisms, and the extension of these findings to complex operational environments.

## Data Availability

The data presented in this study are available on request from the corresponding author.
